# Situs inversus totalis and secondary biliary cirrhosis: a case report

**DOI:** 10.1186/1476-5926-10-5

**Published:** 2011-08-03

**Authors:** Hacı Mehmet Sökmen, Kamil Özdil, Turan Çalhan, Abdurrahman Şahin, Ebubekir Şenateş, Resul Kahraman, Adil Niğdelioğlu, Ebru Zemheri

**Affiliations:** 1 Ümraniye Education and Research Hospital, Department of Gastroenterology, Istanbul, Turkey; 2 Haydarpasa Numune Education and Research Hospital, Department of Gastroenterology, Istanbul, Turkey; 3 Göztepe Education and Research Hospital, Department of Pathology, Istanbul, Turkey

**Keywords:** Situs inversus totalis, secondary biliary cirrhosis, tauroursodeoxycholic acid

## Abstract

Situs inversus totalis is is a congenital anomaly associated with various visceral abnormalities, but there is no data about the relationship between secondary biliary cirrhosis and that condition. We here present a case of a 58 year-old female with situs inversus totalis who was admitted to our clinic with extrahepatic cholestasis. After excluding all potential causes of biliary cirrhosis, secondary biliary cirrhosis was diagnosed based on the patient's history, imaging techniques, clinical and laboratory findings, besides histolopathological findings. After treatment with tauroursodeoxycholic acid, all biochemical parameters, including total/direct bilirubin, alanine aminotransferase, aspartate aminotransferase, alkaline phosphatase and gama glutamyl transferase, returned to normal ranges at the second month of the treatment. We think that this is the first case in literature that may indicate the development of secondary biliary cirrhosis in a patient with situs inversus totalis. In conclusion, situs inversus should be considered as a rare cause of biliary cirrhosis in patients with situs inversus totalis which is presented with extrahepatic cholestasis.

## Background

Situs inversus totalis (SIT) is a congenital anomaly characterized by complete transposition of abdominal and thoracic organs. As a birth defect in newborn infants, it has an estimated incidence of 1/15000 to 10000 cases in live births, with a male/female ratio of 3:2. Generally, this rare anomaly is diagnosed incidentally during thoracic and abdominal imaging. The cause of situs inversus (SI) is unknown. More than one genetic mutations including gene mutations which cause ciliopathy and cystic renal diseases were implicated in etiopathogenesis [1]. SIT is associated with various gastrointestinal abnormalities. In the current literature, development of intestinal ischemia due to intestinal malrotation, and also acute appendicitis and liver transplantation due to juvenile biliary atresia were reported [2-4]. However, there is no data for the development of secondary biliary cirrhosis (SBC) due to extrahepatic cholestasis in a patient with SIT. We here presented a case of SIT with SBC who referred to our clinic due to extrahepatic cholestasis.

## Case presentation

**Figure 1:**
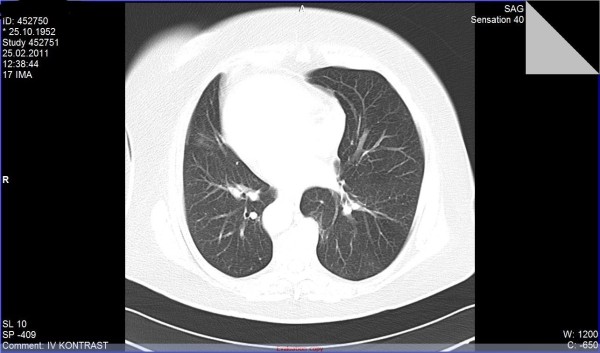
**Thoracic computed tomography scan**. It shows dextrocardia and scars of previous pulmonary infections.

**Figure 2:**
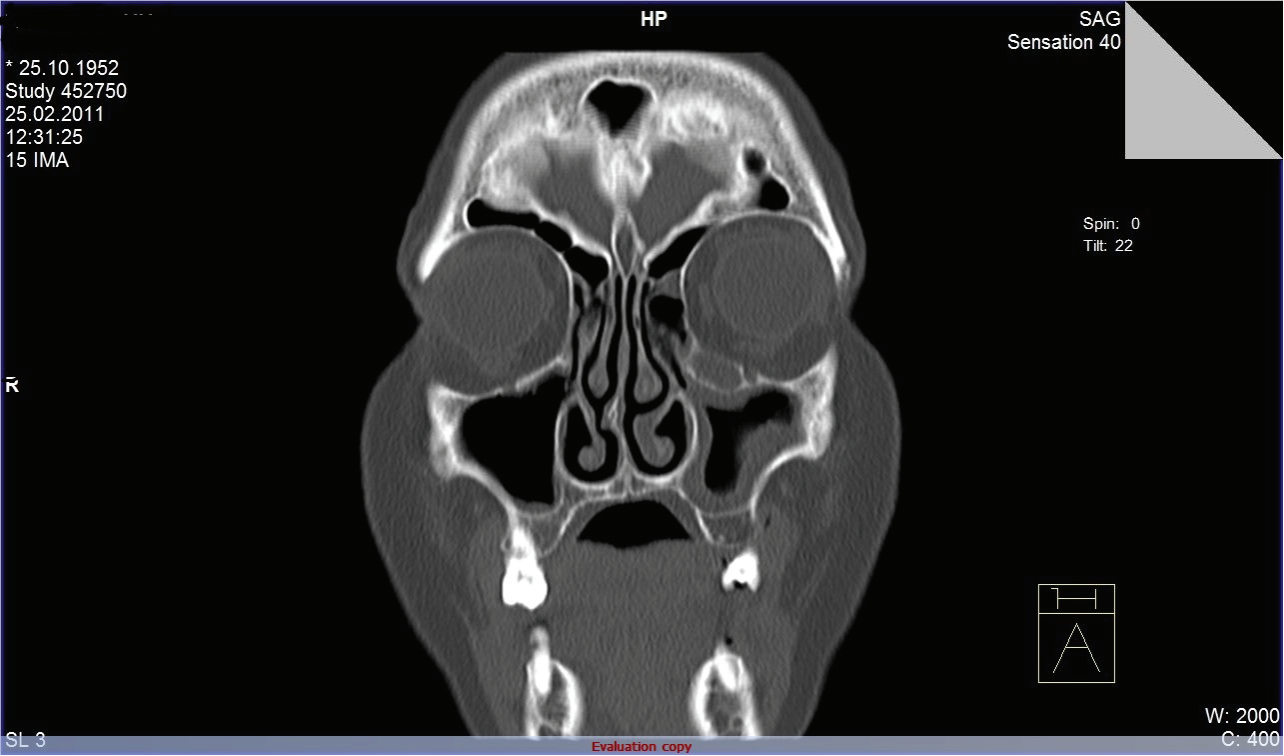
**Paranasal sinus computed tomography scan**. It shows clear chronic sinusitis.

**Figure 3:**
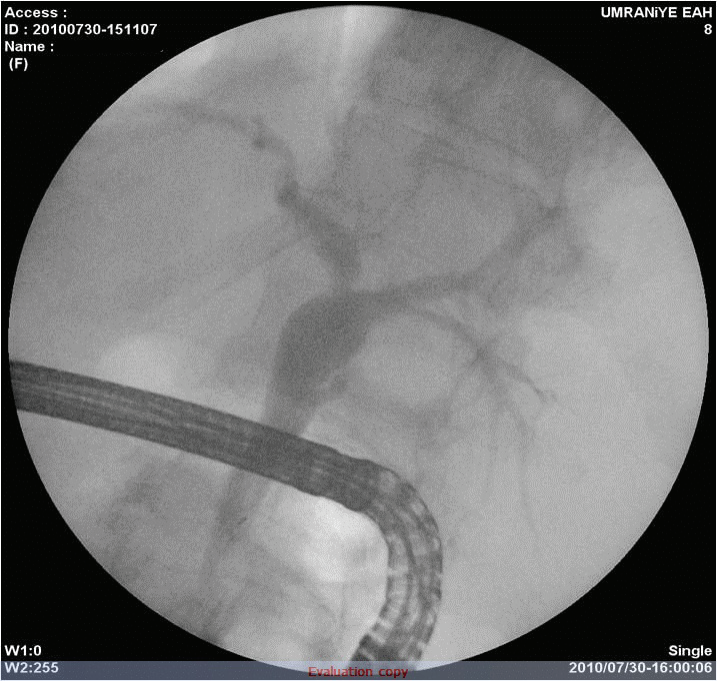
**Endoscopic retrograde colangiopancreatography images**. The choledoc duct is dilated moderately and located on the midline on vertebral axis.

**Figure 4:**
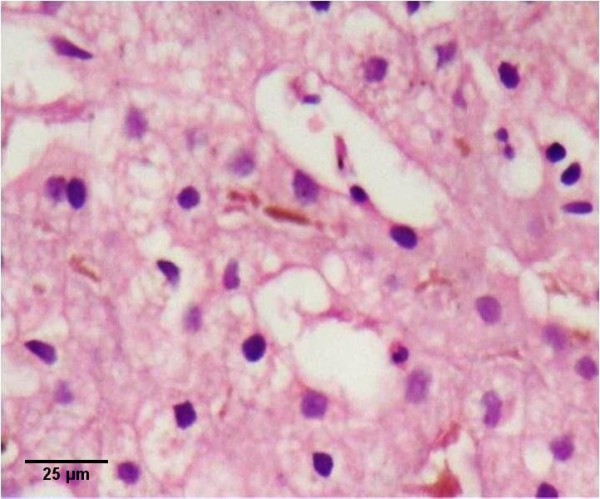
**Canalicular cholestasis, with rosette formation**. Hematoxylin and eosin.

**Figure 5:**
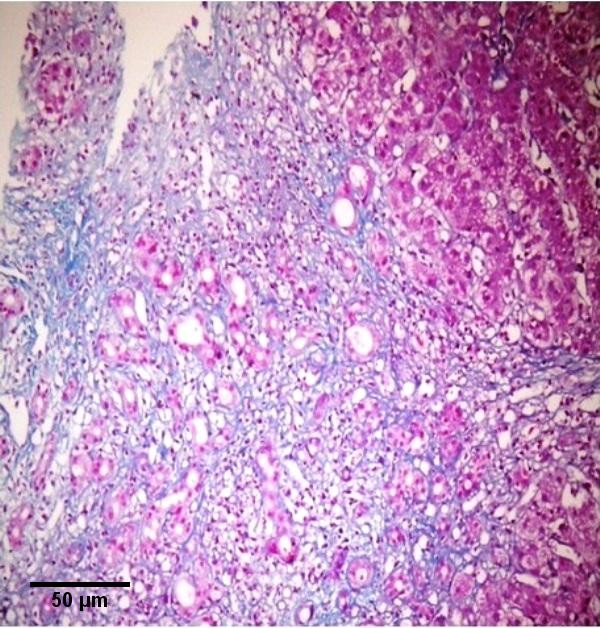
**Portal fibrosis with ductular proliferation**. Masson trichrome.

**Figure 6:**
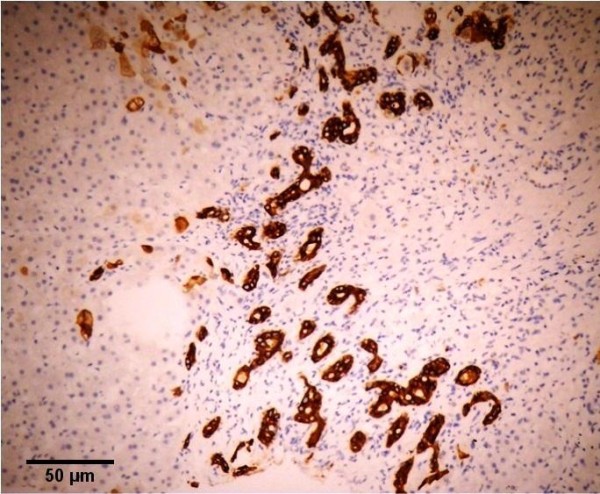
**Ductal and ductular proliferation**. Cytokeratin 7 immunostaining.

A 58-year-old female patient, who complained of icterus appearing in the last 6-7 months, along with the symptoms of fatigue and loss of appetite continued for 2-3 years, was referred to our clinic. According to her medical history, she had been referred to a clinic because of abdominal pain in the left lower quadrant and examined due to acute abdominal pain when she was 6 years old. She had undergone a surgical operation due to acute appendicitis located in the left lower quadrant and the SIT was diagnosed on those days. Furthermore, frequently recurrent upper respiratory tract infections, hypertension and a previous cholecystectomy (19 years ago) were found in her medical history. The patient was a smoker (26 packs/year) but she did not consume alcohol. In detailed personal history, she did not have any hepatotoxic drug usage in past three months. In her physical examination, icteric appearance, moderate hepatomegaly and kyphosis was detected. Her initial laboratory findings were as follows: aspartate aminotransferase (AST) 232 U/L, alanine aminotransferase (ALT) 137 U/L, gama glutamyl transferase (GGT) 252 U/L, alkaline phosphatase (ALP) 153 U/L, bilirubin (total/direct) 22.7/21.4 mg/dl, albumin 2.5 g/dl, leucocyte 8100/mm^3^, hemoglobin 12.5 g/dl, platelet 216000/mm^3^, and INR 1.33. Urea, creatinine and electrolytes were in normal range. In addition, markers of viral hepatitis (anti-HAV IgM, anti-HBc IgM, HBsAg, anti-HCV, TORCH), serology of autoimmune hepatitis (anti-nuclear antibody (ANA), anti-smooth muscle antibody (ASMA), anti-mitochondrial antibody (AMA), liver kidney microsomal antibody (anti-LKM), liver-cytosol spesific antibody (LC-1), anti-soluble liver antigene/liver pancreas (SLA/LP)), transferrine saturation, ferritine and urine copper tests were also in normal ranges. An x-ray of the chest was reported to show dextrocardia. On radiographic image of esophagus and gastric passage, gastric corpus was at the right side of abdominal midline and pylorus and bulbus were located at the left side. In thoracic computed tomography (CT), dextrocardia and scars of previous pulmonary infections were observed (Figure 1). A paranasal sinus CT showed the findings of chronic sinusitis (Figure 2). In transabdominal ultrasonography (US), situs inversus totalis, mild heterogeneous liver parenchyma with grade I hepatosteatosis, choledoc dilatation (11 mm) and mild splenomegaly were determined. Doppler ultrasonography of portal vein revealed a mild splenomegaly and dilated portal vein (14 mm). In endoscopic US, it was noted a choledochal dilatation without stone or sludge and with a diameter of 11.9 mm. In endoscopic retrograde colangiopancreatography (ERCP), performed after pharyngeal local anesthesia and sedation induced with pethidin (50 mg) and i.v. midazolam (5 mg), a dilatation in extrahepatic biliary tracts was observed (Figure 3). Following endoscopic sphincterotomy, extrahepatic biliary tracts were swept by using basket and balloon catheter, but any stone or sludge was not extracted. Since an adequate decrease in cholestasis parameters was not detected after sphincterotomy, a liver biopsy was decided to be performed. In the biopsy material, biliary stasis, rosette formation, feathery degeneration, giant cell formation in lobules, diffuse fibrosis, ductal and ductular proliferation and lymphoplasmocytic infiltration in portal areas were observed (Figures 4, 5 and 6). SBC was diagnosed with patient's history, imaging techniques, clinical and laboratory findings besides histological findings. Thereupon, a 15 mg/kg/day dose of tauroursodeoxycholic acid (TUDCA) was administrated to the patient. During a follow-up period of 9 months, she has been doing well. The laboratory parameters turn to normal ranges in two months and in follow-up period, there was not any abnormal rising in laboratory parameters.

## Conclusions

SI is associated with various gastrointestinal abnormalities such as absence of suprarenal inferior vena cava, polysplenia syndrome, preduodenal portal vein, duodenal atresia or stenosis, tracheoeusophageal fistula (type C), intestinal malrotation, aberrant hepatic arteria, hypoplasia of portal vein, congenital hepatic fibrosis and biliary atresia [5]. In a previous study, it was found that the gallbladder may lie in the midline or be lateralized with the bulk of the hepatic mass [6].

Although the etiology is not clear, it has been suggested that SIT and ciliopathy are related to each other. However, the mechanism has not been explained entirely. It is suggested that the immobility of nodal cilia inhibits the flow of extra embryonic fluid during embryonic period and this leads to SI development [7]. However, primary ciliary dyskinesia (PCD) is observed only in 25% of SI patients.

Whereas a definition of congenital hepatic fibrosis associated with ciliopathy and SIT is reported in the current literature, there is no data about the concurrence of SIT and SBC. Our case is possibly the first one in literature in terms of such SIT and SBC co-existence. Despite there is no clear evident for the development of SBC in patients with SIT, considering the cases reported in literature, the following hypotheses may be proposed. The cilium is a hair like structure that extends from the cell surface into the extracellular space and it has an axoneme containing microtubules, and the microtubules connected with each other with dynein arms that provide ciliary movement [8]. Electron microscopy of the ciliary microtubules frequently reveals absence or abnormalities of the outer and/or inner dynein arms. Especially the mutations of the gene dynein axonemal heavy chain 11 (DNAH 11) are thought to be associated with ciliopathy and SI [9]. From various studies, it was reported that ciliary dyskinesia has a role in the pathogenesis of nephronophthisis (NPHP) and polycystic renal disease (PCD) and the genes that are associated with renal cystic disease are important for left-right axis determination of the body plan [10]. NPHP may be associated with liver fibrosis; patients develop hepatomegaly and moderate portal fibrosis with mild bile duct proliferation, this pattern differs from that of classical congenital hepatic fibrosis, whereby biliary dysgenesis is prominent. Bile duct involvement in cystic kidney disease may be explained by the ciliary theory, because the epithelial cells lining bile ducts (cholangiocytes) possess primary cilia. It was suggested that especially the mutations of the gene NPHP2/inversin is associated with SI. SI and ciliopathy also cause biliary dysgenenesis, dilatation of biliary tract and portal fibrosis [11,12].

In our case, chronic rhinosinusitis and frequently recurrent lower respiratory tract infections, abnormal localization of the main biliary tract (on vertebral axis in ERCP) and moderate dilated biliary tracts support the hypothesis of SIT and ciliopathy association.

There is no data about increased incidence of cholelithiasis in SIT patients. Furthermore, in several case reports, it was suggested that pancreatic ductal carcinoma, autoimmune pancreatitis and sclerosing cholangitis may develop [13,14]. In our patient, there was not any pancreatic pathology. In magnetic resonance cholangiopancreatography (MRCP), ERCP and endoscopic US examinations, there was no finding in favor of cholelithiasis, sclerosing cholangitis or malignity other than moderate choledochal dilatation. Hepatic transaminase enzymes and bilirubin values that were returned to normal ranges with the treatment of a 15 mg/kg/day dose of TUDCA within 2 months supported our diagnosis.

Due to the following reasons, we consider SBC in this case and not primary biliary cirrhosis (PBC): 1) first of all, antimitochondrial antibody was negative in this case; 2) secondly, there was not any symptomatic presentation that seen in PBC such as pruritus, hyperpigmentation, xantalesma; 3) thirdly, in ERCP and MRCP images, choledoc duct was moderately dilated and located on the midline on vertebral axis; 4) finally, it is impossible to differentiate PBC or SBC in such a patient with stage 4 liver fibrosis, but the clinical features and laboratory findings along with histopathological findings supported the SBC. The major causes of SBC are gallstones/choledocholityasis, narrowing of the bile duct following gallbladder surgery, chronic pancreatitis, pericholangitis, idiaptahic sclerosing cholangitis, congenital biliary atresia and cystic fibrosis. In this case, all causes of SBC mentioned above were excluded.

We concluded that this is the first case in literature that may indicate the development of SBC in a patient with SIT.

## Consent

Written informed consent was obtained from the patient for publication of this Case Report. A copy of the written consent is available for review by the Editor-in-Chief of this journal.

## Competing interests

The authors declare that they have no competing interests.

## Authors' contributions

HMS carried out endoscopic ultrasonography (EUS) and participated in coordination and drafted the manuscript. KÖ carried out the endoscopic retrograde cholangiopancreaticography (ERCP), TÇ conceived of the case report, and participated in its design and coordination and helped to draft the manuscript. AŞ helped collecting the data of the patient. EŞ conceived of the case report, and participated in its design and coordination and helped to draft the manuscript. RK and AN followed the patients after externalization to date. EZ assessed the pathological materials of the patient. All authors read and approved the final manuscript.
